# Nonalcoholic Fatty Liver Disease: Pros and Cons of Histologic Systems of Evaluation

**DOI:** 10.3390/ijms17010097

**Published:** 2016-01-13

**Authors:** Elizabeth M. Brunt

**Affiliations:** Department of Pathology and Immunology, Washington University School of Medicine, Campus Box 8118, St. Louis, MO 63110, USA; ebrunt@wustl.edu; Tel.: +314-747-0143

**Keywords:** nonalcoholic fatty liver disease, nonalcoholic steatohepatitis, pathology

## Abstract

The diagnostic phenotype of nonalcoholic fatty liver disease (NAFLD)—in particular, the most significant form in terms of prognosis, nonalcoholic steatohepatitis (NASH)—continues to rely on liver tissue evaluation, in spite of remarkable advances in non-invasive algorithms developed from serum-based tests and imaging-based or sonographically-based tests for fibrosis or liver stiffness. The most common tissue evaluation remains percutaneous liver biopsy; considerations given to the needle size and the location of the biopsy have the potential to yield the most representative tissue for evaluation. The pathologist’s efforts are directed to not only global diagnosis, but also assessment of severity of injury. Just as in other forms of chronic liver disease, these assessments can be divided into necroinflammatory activity, and fibrosis with parenchymal remodeling, in order to separately analyze potentially reversible (grade) and non-reversible (stage) lesions. These concepts formed the bases for current methods of evaluating the lesions that collectively comprise the phenotypic spectra of NAFLD. Four extant methods have specific applications; there are pros and cons to each, and this forms the basis of the review.

## 1. Introduction

The value of liver biopsy evaluation for diagnosis in clinical care and effectiveness of intervention in clinical research in the field of nonalcoholic fatty liver disease (NAFLD) has remained unquestioned as knowledge in the field has continued to grow over the course of the last three and a half decades since the publication attributed as one of the early descriptions in humans [1]. Currently several clinical algorithms based on serum-based tests can be used to predict the likelihood of NAFLD, nonalcoholic steatohepatitis (NASH) or presence or severity of fibrosis, reviewed [2]. As well, sonographically-based tests of liver “stiffness” and imaging-based tests for presence of hepatic fat are variably validated and becoming more available [3]. The unquestioned value of all non-invasive testing is for patient follow-up; in sophisticated hands, these tests also play a role in determination of need for liver biopsy, as the latter, an invasive test with known low but potential risk of morbidity cannot be utilized as a screening test [4]. The best noninvasive tests have been developed and validated against the “gold-standard” of liver biopsy in order to produce equivalent information regarding the state of the liver parenchyma.

Liver biopsy cannot be considered a “perfect” test, however, but the short-comings of this can largely be overcome once understood. For instance, the consideration of sampling “error” [5] was detailed in a study in 2005 that demonstrated differences in grade and stage by the blinded pathologist even when biopsies were obtained from the identical location. However, as in most chronic liver diseases, this “error” is likely a reflection of the disease heterogeneity of NAFLD, and must be accounted for by providing sufficient numbers of subjects in clinical trials. Another less well-known short-coming of liver biopsy, particularly when done by radiologists, or in the setting of bariatric surgery, is the use of appropriately sized (*i.e.*, large-bore) needles, [6], and potential differences between the right and left lobes of the liver. For instance, the subcapsular portal tracts in the left lobe are larger and closer to the capsule than in the right lobe; if not aware of this, a pathologist can misinterpret the seemingly enlarged portal structures from a left lobe biopsy for fibrotic portal structures, particularly if a small bore needle has been used to obtain the biopsy. Determining histologic inflammation in the liver parenchyma will not lead to valid results from a biopsy obtained in a surgical procedure, as anesthesia alone will lead to parenchymal and perivenular collections of polymorphonuclear leukocytes, collectively known as “surgical hepatitis”. Discerning which foci were present prior to anesthesia, and which are due to surgical hepatitis is not possible. Further, if a study protocol includes biopsy, agreement of exact location should be made in advance with all investigators so that pre and post intervention biopsies are truly comparable. Finally, the interpreting pathologist’s expertise and familiarity with the spectra of lesions in the disease process are factors to be considered in NAFLD, as in any other form of liver disease [7,8].

Once the decision for liver biopsy has been made, whether for clinical (*i.e.*, diagnostic or prognostic) purposes, or for clinical trial protocol, the next steps involve the histopathologic interpretation for diagnosis, and for semi-quantitative lesion evaluations, if requested, for protocol or study purposes. Methods for these are the subjects of the remainder of this review.

## 2. Diagnosing Fatty Liver Disease in Liver Biopsy

Before any form of assessment of severity of injury or fibrosis can be applied, the pathologist must be certain that the biopsy actually is diagnostic of the clinically presumptive disease; this basic exercise applies to all forms of liver disease. NAFLD is an umbrella term applied to a range of histopathologic phenotypes in adults, adolescents and children. It is important that the pathologist report is limited to the findings noted, and count on the clinical team to put these together with all information regarding possible etiologies that may present in a similar manner, including, for instance, Wilson disease, other inborn errors of metabolism, and alcoholic liver disease. Discussed in detail in recent reviews [9], they will be briefly summarized herein. In adults, prior to advanced fibrosis and parenchymal remodeling (nodularity), the parenchyma shows varying degrees of steatosis within the zone 3 hepatocytes (those around the terminal hepatic venule). The large and small droplet steatosis is termed macrovesicular due to the fact it is either a single large droplet or several droplets readily separable to the microscopic eye. Often, the two are co-existent in the same hepatocyte. Thus, the term, “large and small droplet macrovesicular steatosis” is applied. When only steatosis is present in the biopsy, the diagnostic term, NAFL, is given. For this, >5% of hepatocytes within the biopsy must be occupied by this type of visible fat droplets. In a minority of cases, non-zonal clusters of hepatocytes also have true microvesicular steatosis; an association was noted with greater severity of disease in these cases in a large series from the NASH Clinical Research Network (CRN) [10]. The terminal “D” of NAFLD is removed by convention, as steatosis is considered non-progressive, although exceptions have been noted in subjects, most of whom subsequently gain weight or the metabolic status changed [11,12].

The second component of NAFLD is inflammatory cells; these may be seen within the acini (aka lobules), or in portal tracts, or both. The inflammatory components of this disease are quite complex, but with the routine hematoxylin and eosin stain to the pathologist’s view microscopically, can be divided into mononuclear cells (lymphocytes, monocytes), eosinophils, polymorphonuclear cells (pmn’s), and Kupffer cells. Even occasional plasma cells can be noted. Kupffer cells are pigmented, enlarged and either singly or in clusters surrounding an apoptotic hepatocyte (microgranuloma) or a fat droplet (lipogranuloma). Lipogranulomas often have an associated eosinophilic leukocyte, and when adjacent to a terminal hepatic venule or within a portal tract, may also have collagen. Epithelioid or caseating granulomatous inflammation are not features of NAFLD, and deserve further attention. Pmn’s surrounding individual hepatocytes, referred to as “satellitosis”, are indicative of alcoholic hepatitis; clusters of pmn’s signify possible sepsis or may occur if the biopsy is obtained during a surgical procedure when the patient is under anesthesia. Thus, caution is warranted when pmn clusters are easily noted; attempting to “count” inflammatory foci in such a specimen is not advisable.

Portal inflammation consists of similar cell types as described above (except the macrophages are not Kupffer cells) in varying degrees, including lipogranulomas. Other types of granulomatous inflammation should be further evaluated. Bile duct injury may be seen, but should be further evaluated. Marked portal inflammation and lymphoid aggregates, diffuse interface activity, and plasma-cell rich infiltrates are all lesions that deserve further investigation. Numerous polymorphonuclear leukocytes, when present, are typically present as cholangitis, cholangiolitis/pericholangitis and indicate an extra-hepatic biliary process such as obstruction or pancreatitis, or alcoholic liver disease. Canalicular bile plugs in zone 3 correlate with these findings and warrant further investigation. Cholangiolar bile is indicative of sepsis. The combination of macrovesicular steatosis and inflammation has been termed steatosis with inflammation; this is not, however, diagnostic of steatohepatitis.

### 2.1. Nonalcoholic Steatohepatitis (NASH)

For the diagnosis of NASH, the most recognized form of injury in NAFLD with potential to progress to fibrosis and cirrhosis and its complications, there is a requirement not only for the steatosis and inflammation as described above, but also for a particular form of hepatocyte injury known as ballooning. While some authorities have stated that steatosis with inflammation and perisinusoidal fibrosis are adequate for a diagnosis of steatohepatitis, it is not clear that this group of findings represents a lesion with actual potential of progression, or represents a step in regression of steatohepatitis (*i.e.*, loss of ballooning). The NASH CRN Pathology Committee categorizes this within a set of lesions as “Borderline, Zone 3”, and specifies that hepatocellular ballooning must be present for a diagnosis of steatohepatitis. NASH can be diagnosed in the absence of fibrosis. The initial collagen deposition in adult NASH is in the perisinusoidal spaces in zone 3; with progression, fibrosis is additionally noted in periportal spaces, often associated with a ductular reaction. More advanced fibrosis is indicated by bridging between vascular structures: central veins to central veins (via perisinusoidal spaces); central–portal; portal–portal; with nodularity of the intervening parenchyma. Cirrhosis is the final outcome of advanced fibrosis and remodeling. Residual perisinusoidal fibrosis may or may not remain.

An intriguing and important concept in NASH is that with advanced disease, *i.e.*, fibrosis and architectural remodeling with bridging fibrosis and nodularity, and ultimately the vascular remodeling of cirrhosis, the lesions of activity described above may or may not continue to be present. Investigators have used this information to justify a correlation with the assignment of the diagnosis of “cryptogenic cirrhosis” to cases in which no identifying lesions of active liver disease can be found. In a strict sense, however, without a prior biopsy diagnosis of NASH, this may not be correct in all cases. Many cases of cryptogenic cirrhosis, in fact, may be burned-out cirrhosis from other causes such as prior alcohol abuse, autoimmune hepatitis, heterozygous α-1-antitrypsin liver disease, or even more rare processes (e.g., keratin mutations). However, there are bona fide cases of burned-out NASH in which there remain histologic “hints”: e.g., foci of perisinusoidal fibrosis, occasional ballooned hepatocytes, rare Mallory–Denk bodies in a non-alcohol user. If there is a prior biopsy with NASH, the “burned-out” cirrhosis is no longer “cryptogenic”, but is cirrhosis secondary to prior NASH.

### 2.2. Pediatric Nonalcoholic Fatty Liver Disease (NAFLD)

Pediatric NAFLD is known to be unique in its pre-cirrhotic histopathologic features. This has been accepted since the seminal descriptions of Schwimmer *et al.* in 2005 [13], and validated subsequently by others. Interestingly, as of yet, there is no accepted diagnosis of “steatohepatitis” in children, although clearly the end results, cirrhosis and hepatocellular carcinoma, do occur. The initial findings in children are of large droplet macrovesicular steatosis either in a periportal or panacinar distribution and when inflammation is present, it is more common in the portal collagen than in the lobules. Ballooned hepatocytes are few if any. Portal expansion by fibrosis occurs initially, and perisinusoidal fibrosis may or may never be seen. The categorization of Borderline, Zone 1 has been utilized by the NASH CRN for the above-described lesions.

## 3. Grading and Staging the Lesions of NAFLD

Four current methods of semi-quantitatively evaluating histologic lesions of NAFLD are summarized in Table 1; they include a proposal referred to as the “Brunt” system [14], the NASH CRN Pathology Committee system for NAFLD Activity Score (NAS) and fibrosis score [15], the “Fatty Liver Inhibition of Progression (FLIP)” algorithm [16,17] and a pediatric score based on weighted values for the features of NAFLD, the Pediatric NAFLD Histologic Score [18]. The first was restricted to adults and to NASH; the middle two can apply to the full range of NAFLD; the NASH CRN system alone applies to adults and children.

### 3.1. Brunt Proposal for Grading and Staging

The proposal for grading and staging the lesions of NASH was made when the disease itself was still being questioned as an entity other than surreptitious alcoholism; it was clear that further work would not progress until a systematic method of analyzing the pathology was in place. Thus, this proposal was just that: a first proposed method to separately analyze grade and stage, similarly to what was being done with other forms of chronic hepatitis, but with adjustments for the lesions of fatty liver disease [14]. There was systematic review of 52 adult biopsies from 51 clinically diagnosed subjects with NASH with semi-quantitative assessment and notation of location of steatosis, and ballooning; semi-quantitative assessment for lobular and portal inflammation and Periodic Acid Schiff after diastase digestion (PASd) Kupffer cells, Mallory–Denk bodies, acidophil bodies, iron, and glycogenated nuclei, lipogranulomas and locations of fibrosis, zone 3 perisinusoidal, portal/periportal, and bridging. “Gestalt” diagnosis of severity of each case (mild, moderate, severe) then followed. The “global grade” was based on review of the semi-quantitative lesions and impression-based grades, and focused in particular on steatosis, hepatocellular ballooning, zone 3 accentuation of injury. It was noted that the initial, and often persistent form of fibrosis is perisinusoidal; this differs from the distinctly portal-based fibrosis of chronic hepatitis and biliary fibrosis. The so-called “Brunt” method continued the paradigm of maintaining separation of grade (lesions of activity) and stage (lesions of fibrosis and parenchymal remodeling), as had been established by the systems for evaluation in chronic hepatitis [19]. The method of grading and staging was written to be applied after the diagnosis of NASH had been rendered, and was considered a “global” assessment such that grades 1–3, mild, moderate and severe, were evaluations of combinations of steatosis, lobular and portal inflammation and ballooning. Hepatocyte ballooning was noted as the major determinant of severity and steatosis amount was the least determinant; inflammation increased with each grade. Fibrosis was scored according to the observed location and extent of collagen deposition as described above. Grade and stage were noted to be disparate, as in chronic hepatitis although none of the low stage biopsies showed severe steatohepatitis. Higher grade did correlate with higher mean aspartate aminotransferase (AST), but not with alanine aminotransferase (ALT). This system was created for NASH, and thus did not take into account the full spectrum of NAFLD, nor did the system address lesions of pediatric NAFLD. Although the system has been widely utilized and applied, it was never formally validated. It is, however, a useful benchmark for diagnosing NASH as it highlighted the increasing severity of ballooning with increased severity of grade. This proposal also documented the characteristic fibrosis of adult NASH.

**Table 1:** Histologic methods of semi-quantitative evaluation of nonalcoholic fatty liver disease (NAFLD).

System/ Characteristics	Brunt System [14]	NASH CRN [15]	* SAF/** FLIP Algorithm [16,17]	Pediatric NAFLD Histologic Score [18]
Patient Population	Adults only	Adults + Children	Adults	Children
Applicable to	NASH	All NAFLD	All NAFLD	Peds NAFLD
Grade	Mild, Moderate, Severe; S + LI, PI + B; Unweighted but steatosis does not affect score; LI + PI, ballooning increase incrementally with score	NAFLD Activity Score (NAS): S + LI + B = 0–8; Unweighted scores for each lesion	Steatosis is not a component of activity Activity: LI + B; * SAF: Steatosis + Actitivity + Fibrosis = S_x_A_x_F_x_; ** FLIP: Fatty Liver Inhibition of Progression algorithm for diagnosis	Weighted sums of S + LI + PI + B, see text
Details of Scoring	Steatosis	Steatosis	Steatosis	Same as NASH CRN plus Portal Inflammation 0–2
	0: 0	0: <5%	0: <5%	
	1: 0%–33%	1: 5%–33%	1: 5%–33%	
	2: 34%–66%	2: 34%–66%	2: 34%–66%	
	3: >66%	3: >67%	3: >67%	
	LI	LI	LI	
	0:0	0:0	0: 0	
	1: 1–2/20x	1: <2/20x	1: <2/20x	
	2: 2–4/20X	2: 2–4/20X	2: 2/20x	
	3: >4/20X	3: >4/20X	–	
	PI:	Ballooning:	Ballooning: 0–2	
	0: none	0: None	0: 0	
	1: mild	1: Few	1: clusters, reticulated cytoplasm	
	2: moderate	2: Many	2: enlarged hepatocytes	
	3: severe	–	–	
	Ballooning	Prominent	–	
	Acinar location	–	–	
	Mild	–	–	
	Marked	–	–	
	Fibrosis Stage	Fibrosis Stage	Fibrosis Stage	Fibrosis Stage: As with NASH CRN
	0: none	0: none	F0: 0	
	1: zone 3 perisinusoidal	1a: zone 3 perisinusoidal, delicate	F1: zone 3 perisinusoidal (all), or portal only	
		1b: zone 3 perisinusoidal, dense		
		1c: portal only		
	2: 1 + periportal	2: 1a or 1b + periportal	F2: zone 3 + periportal	
	3: bridging	3: bridging	F3: bridging	
	4: cirrhosis	4: cirrhosis	F4: cirrhosis	
Fibrosis Stages	0–4	0–4; # 1a, 1b, 1c	0–4; # 1a, 1b, 1c	0–4
Scoring Method Used for diagnosis	Minimal criteria for dx	Correlates but does not replace; used in clinical trials for feature comparisons	Yes, for diagnosis	Yes, for diagnosis
Clinical Associations	Grade: AST	ALT, AST	AST, ALT	WC, MetSynd, TG, Fibrosis in biopsy

S = steatosis amount; LI = lobular inflammation; PI = portal inflammation; B = ballooning; # 1a, 1b, 1c: see text. ALT = alanine aminotransferase; AST = aspartate aminotransferase; WC = Waist circumference; MetSynd = Metabolic Syndrome; TG = triglyceride levels.

### 3.2. NASH Clinical Research Network (CRN) Scoring System

The National Institute of Digestive Diseases and Kidney (NIDDK) of the National Institutes of Health (NIH) established the NASH Clinical Research Network (CRN) in order to undertake multicenter observational and interventional trials. The Pathology Committee was tasked with developing and validating a method for semi-quantitatively evaluating changes in histologic features in these studies. The result was a feature-based system referred to commonly as the NAFLD Activity Score (NAS) [15]. This is a score for lesions of activity based on carefully analyzed results of 32 twice-reviewed biopsies of adults and 18 once reviewed biopsies of children by a group of 9 liver pathologists. The review consisted of 14 lesions of NAFLD (the same as above in similar fashion, plus presence of foci of microvesicular steatosis, megamitochondria, and microgranulomas) and three diagnostic categories: NASH, not NASH and borderline. The lesions that ultimately comprised the NAS were determined by multiple logistic regression to correspond with the separately derived diagnoses of NASH: macrovesicular steatosis, lobular inflammation and ballooning. The final NAS was based on unweighted scores of each, and ranged from 0 to 8. As noted in Table 1, although the lesion scores are unweighted, the fact that steatosis and lobular inflammation range from 0 to 3 whereas ballooning range from 0 to 2, gives steatosis more weight in the NAS. The separately derived diagnoses of NASH mostly correlated with scores ≥5; NAS < 3 had been diagnosed as not NASH. Fibrosis stage was a modification of the Brunt system in order to account for pediatric portal-only fibrosis (stage 1c); zone 3 delicate (1a) or dense (1b) fibrosis were created for the purpose of clinical trials. The manuscript that presented the NAS described other observations of importance that remain relevant today: the diagnosis does not rest solely on the presence of particular lesions; the score was not created to replace a pathologist’s diagnosis or as a severity scale or to measure rapidity of progression, but rather as a method of analysis in assessing overall histologic change. A subsequent study of 976 centrally reviewed adult biopsies from the NASH CRN highlighted the significance of separating the pattern-based pathologists’ activity of diagnosis from the feature-based score [20]. Although there was significant overlap between the diagnosis and the NAS, some details are worth re-iterating. While 75% of definite steatohepatitis cases had NAS ≥ 5, 28% of borderline diagnoses and 7% of “not NASH” also had NAS ≥ 5. Thus, for clinical trial entry, or for clinical management, if the NAS were the basis of decision making, the latter and last cases would be “mis-categorized”. Further, and of most importance, in a regression model, while both the diagnosis of steatohepatitis and the NAS were statistically strongly associated with liver enzymes (ALT and AST) in both the one variable (either NAS or NASH diagnosis) and two variable (both NAS and NASH diagnosis) models, and features of Metabolic Syndrome, diabetes, and measures of insulin resistance, the homeostatic model assessment of insulin resistance and the quantitative insulin sensitivity check index (HOMA-IR and QUICKI) were associated with both in the one variable model, these latter features only remained statistically associated with the diagnosis of steatohepatitis in the two variable model. Thus, the implication is strong that not only are the particular histologic features of steatohepatitis important, but the overall pattern of those features (*i.e.*, the determination of diagnosis) is important in correlation with liver injury, as well as underlying factors of the disease process.

### 3.3. Fatty Liver Inhibition of Progression (FLIP) Algorithm

A third approach to adult NAFLD scoring has been proposed and validated by Bedossa *et al.* [17]; the score was developed in 679 liver biopsies from morbidly obese patients undergoing bariatric surgery with at least one metabolic complication (*i.e.*, diabetes, hypertension, dyslipidemia or obstructive sleep apnea), and validated in 60 liver biopsies of subjects with metabolic syndrome, but not morbid obesity. The algorithm, subsequently tested for observer variability by two groups of pathologists, a European study group, the Fatty Liver Inhibition of Progression (FLIP) pathology group, and a pathology group of general pathologists with varying amounts of liver pathology training [16]. The score is based on two now recognized concepts; even though large droplet macrovesicular steatosis is an obviously recognized, and required, feature of non-cirrhotic NAFLD, it is likely not a driver in progression of disease, thus, the feature should not carry much weight, if any at all, in a histologic score. However, ballooning and lobular inflammation have been noted in several studies to be significant features in progressive disease, thus, these should be more weighted as determinants of progression. Thus, the “activity score” is derived from the combination of the semi-quantitative values of the two [17]. The details for semi-quantitative scores differ slightly from prior methods: lobular inflammation ranges from 0 to 2 (instead of 0–3), ballooning 0–2 (with descriptions of ballooning as detailed in Table 1). As the final score is meant to represent a diagnosis, steatosis (S_x_) must be >0, activity (ballooning plus lobular inflammation (A_x_) must be ≥2, in which ballooning is at least 1. Fibrosis is based on the NASH CRN scale, and reported as “F”. One of the primary advantages of this score is the manner of reporting: by giving a subscore for each component of the SAF (Steatosis + Activity + Fibrosis), the amount of steatosis and fibrosis are communicated and one may make comparisons for the features with other biopsies from the same patient. Activity, the most important of the scores is an additive score, so, similar to the NAS, one cannot determine how much is ballooning and how much is lobular inflammation, thus, as with the NAS, improvement in either would not be visible by the SAF alone. Increased values of the SAF correlated well with increased values of serum AST and ALT. Correlations with known metabolic features of NAFLD/NASH, such as markers of insulin resistance, were not reported for the different activity scores that discriminate NAFLD and NASH.

The second study done by the FLIP pathologists and a group of community pathologists [16] was done to test the validity of the SAF algorithm in non-bariatric subjects as well as to test the usefulness of such an algorithm for practical use. Both groups of pathologists’ diagnoses improved when the SAF was utilized and both groups had high kappa values when utilizing the SAF. One of the discussed concepts was the challenge for pathologists to make the distinction(s) of NASH and non-NASH in liver biopsy material, whereas use of an algorithm such as the SAF could mitigate against the necessity of such. An example given was a case of steatosis with only fibrosis, but no other features. Additionally, the graphic of the SAF score showed that it could be possible to have S > 0 A_1(B1 + L0)_ (*i.e.*, steatosis > 0, activity score of 1 because of ballooning score of 1 but no lobular inflammation) with a final diagnosis of “steatosis”. Both of these examples are troublesome and highlight the oversimplification of the SAF on its own. The former could potentially fit into a “borderline” category of either zone 1 or zone 3 depending on where the fibrosis is located and the latter could fit into borderline zone 3, also depending if the ballooning and steatosis were in zone 3. Alternatively, both could fit into examples of resolution of prior NASH, and one would want to compare them with prior biopsies. Although both of the studies that proposed and discussed the values of the SAF reiterated that it was not meant to replace a written pathology report, neither mentioned the authors’ concepts of fundamentals of NASH diagnosis other than the presence of the lesions in the SAF. Zonal localization and accentuation of lesions in adults and children were not assessed, nor can they be, by the algorithm proposed.

### 3.4. Pediatric NAFLD Histologic Score

The final scoring system proposed is specifically for the pediatric group by Alkhouri *et al.* [18]. The score proposed was developed from 203 biopsies of children with NASH or “notNASH” according to the pathologist’s diagnosis, and given NAS and fibrosis scores according to the NIDDK NASH CRN system with the exception of adding a portal inflammation score of 0–2 for none, mild or moderate portal inflammation. After logistic regression, each feature was weighted and a final Pediatric NAFLD Histologic Score (PNHS) was developed that can be calculated by entering their values into the website [21]. Both the training and validation sets had high area under receiver operating curve (AUROC) values. Interestingly, 65.9% of NASH biopsies had ballooning, as did 4.4% of notNASH biopsies, but 34.1% of NASH biopsies also were diagnosed as such without ballooning. The NAS was greater in NASH biopsies than in notNASH biopsies (mean values 4.5 ± 1.4 *vs.* 2.2 ± 0.59, *p* < 0.001), as expected, as was fibrosis >0 (*p* < 0.001). The score was developed in order to better reflect pediatric “NASH” than the term “borderline” steatohepatitis for both clinical care and clinical trials. Whether it has been in use long enough to accomplish this goal or not cannot be clearly stated at this time. The need to utilize a website for determination of a score and therefore a diagnostic category is interesting and the goal worthwhile, but the concept is somewhat worrisome to diagnostic pathologists as the suggestion that a calculated algorithm can actually replace the interpretative experience that is involved in deriving a final diagnosis is not something one accepts with certainty. The “art” of interpretation continues to play a role in all fields of medicine, regardless of the rigor with which it is applied.

## 4. Conclusions

It is apparent that NAFLD and NASH are complex entities, not only for clinicians, basic scientists, but also for diagnostic pathologists. Even though much progress has been made, it is worthwhile to remember that scoring methods are measures of injury, but not replacements of diagnostic assessment, and thus, pathologists need to first be trained to recognize patterns of disease, and then to apply appropriate scoring systems. There are pros and cons to any scoring system for all disease processes, as discussed above for NAFLD and NASH. As continued work is done, however, the expectations for more “pros” and fewers “cons” remain.

## References

[1] Ludwig J., Viggiano T.R., McGill D.B., Oh B.J. (1980). Nonalcoholic steatohepatitis: Mayo Clinic experiences with a hitherto unnamed disease. Mayo Clin. Proc..

[2] Brunt E.M., Neuschwander-Tetri B.A., Burt A.D., Burt A.D., Portmann B., Ferrell L. (2012). Fatty Liver Disease: Alcoholic and Nonalcoholic. MacSween’s Pathology of the Liver.

[3] Brunt E.M., Wong V.W.-S., Nobili V., Day C.P., Sookoian S., Maher J.J., Sirlin C., Neuschwander-Tetri B.A., Rinella M.E. (2015). Nonalcoholic fatty liver disease. Nat. Rev. Prim..

[4] Torres D.M., Williams C.D., Harrison S.A. (2012). Features, diagnosis, and treatment of nonalcoholic fatty liver disease. Clin. Gastroenterol. Hepatol..

[5] Ratziu V., Charlotte F., Heurtier A., Gombert S., Giral P., Bruckert E., Grimaldi A., Capron F., Poynard T. (2005). Sampling variability of liver biopsy in nonalcoholic fatty liver disease. Gastroenterology.

[6] Larson S.P., Bowers S.P., Palekar N.A., Ward J.A., Pulcini J.P., Harrison S.A. (2007). Histopathologic variability between the right and left lobes of the liver in morbidly obese patients undergoing Roux-en-Y bypass. Clin. Gastroenterol. Hepatol..

[7] Vuppalanchi R., Unalp A., van Natta M.L., Cummings O.W., Sandrasegaran K.E., Hameed T., Tonascia J., Chalasani N. (2009). Effects of liver biopsy sample length and number of readings on histologic yield for nonalcoholic fatty liver disease. Clin. Gastroenterol. Hepatol..

[8] Bedossa P., Bioulacsage P., Callard P., Chevallier M., Degott C., Deugnier Y., Fabre M., Reynes M., Voigt J.J., Zafrani E.S. (1994). Intraobserver and interobserver variations in liver biopsy interpretation in patients with chronic hepatitis c. Hepatology.

[9] Kleiner D.E., Brunt E.M. (2012). Nonalcoholic fatty liver disease: Pathologic patterns and biopsy evaluation in clinical research. Semin. Liver Dis..

[10] Tandra S., Yeh M.M., Brunt E.M., Vuppalanchi R., Cummings O.W., Unalp-Arida A., Wilson L.A., Chalasani N. (2011). Presence and significance of microvesicular steatosis in nonalcoholic fatty liver disease. J. Hepatol..

[11] Pais R., Charlotte F., Fedchuk L., Bedossa P., Lebray P., Poynard T., Ratziu V. (2013). A systematic review of follow-up biopsies reveals disease progression in patients with non-alcoholic fatty liver. J. Hepatol..

[12] McPherson S., Hardy T., Henderson E., Burt A.D., Day C.P., Anstee Q.M. (2015). Evidence of NAFLD progression from steatosis to fibrosing-steatohepatitis using paired biopsies: Implications for prognosis and clinical management. J. Hepatol..

[13] Schwimmer J.B., Behling C., Newbury R., Deutsch R., Nievergelt C., Schork N.J., Lavine J.E. (2005). Histopathology of pediatric nonalcoholic fatty liver disease. Hepatology.

[14] Brunt E.M., Janney C.G., di Bisceglie A.M., Neuschwander-Tetri B.A., Bacon B.R. (1999). Nonalcoholic steatohepatitis: A proposal for grading and staging the histological lesions. Am. J. Gastroenterol..

[15] Kleiner D.E., Brunt E.M., van Natta M., Behling C., Contos M.J., Cummings O.W., Ferrell L.D., Liu Y.C., Torbenson M.S., Unalp-Arida A. (2005). Design and validation of a histological scoring system for nonalcoholic fatty liver disease. Hepatology.

[16] Bedossa P. (2014). Utility and appropriateness of the fatty liver inhibition of progression (FLIP) algorithm and steatosis, activity, and fibrosis (SAF) score in the evaluation of biopsies of nonalcoholic fatty liver disease. Hepatology.

[17] Bedossa P., Poitou C., Veyrie N., Bouillot J.-L., Basdevant A., Paradis V., Tordjman J., Clement K. (2012). Histopathological algorithm and scoring system for evaluation of liver lesions in morbidly obese patients. Hepatology.

[18] Alkhouri N., de Vito R., Alisi A., Yerian L., Lopez R., Feldstein A.E., Nobili V. (2012). Development and validation of a new histological score for pediatric non-alcoholic fatty liver disease. J. Hepatol..

[19] Brunt E.M. (2000). Grading and staging the histopathological lesions of chronic hepatitis: The Knodell histology activity index and beyond. Hepatology.

[20] Brunt E.M., Kleiner D.E., Wilson L., Belt P., Neuschwander-Tetri B.A. (2011). NASH Clinical Research Network (CRN). The NAS and the histopathologic diagnosis of NASH: Distinct clinicopathologic meanings. Hepatology.

[21] Pediatric NAFLD Histologic Score. http://rcc.simpal.com/RCEval.cgi?RCID=RPCxtv#RESULT.

